# Epidemiology and patterns of empiric antimicrobial therapy practice in patients with community-onset sepsis using data from a Japanese nationwide medical claims database—the Japan Sepsis Alliance (JaSA) study group

**DOI:** 10.1016/j.ijregi.2024.01.002

**Published:** 2024-01-03

**Authors:** Toshikazu Abe, Hiroki Iriyama, Taro Imaeda, Akira Komori, Takehiko Oami, Tuerxun Aizimu, Nozomi Takahashi, Yasuo Yamao, Satoshi Nakagawa, Hiroshi Ogura, Yutaka Umemura, Asako Matsushima, Kiyohide Fushimi, Nobuaki Shime, Taka-aki Nakada

**Affiliations:** 1 Department of Emergency and Critical Care Medicine, Tsukuba Memorial Hospital, Tsukuba, Japan; 2 Health Services Research and Development Center, University of Tsukuba, Tsukuba, Japan; 3 Department of Health Services Research, Faculty of Medicine, University of Tsukuba, Japan; 4 Department of Emergency and Critical Care Medicine, Chiba University Graduate School of Medicine, Chiba, Japan; 5 Department of Critical Care Medicine, National Center for Child Health and Development, Tokyo, Japan; 6 Department of Traumatology and Acute Critical Medicine, Osaka University Graduate School of Medicine, Osaka, Japan; 7 Department of Emergency & Critical Care, Nagoya City University Graduate School of Medical Sciences, Aichi, Japan; 8 Department of Health Policy and Informatics, Tokyo Medical and Dental University Graduate School of Medical and Dental Sciences, Tokyo, Japan; 9 Department of Emergency and Critical Care Medicine, Graduate School of Biomedical, and Health Sciences, Hiroshima University, Hiroshima, Japan

**Keywords:** Sepsis, Anti-infective agents, Infections, Community-onset sepsis, Antibacterial agents

## Abstract

**Objectives:**

We aimed to describe empiric antimicrobial options for patients with community-onset sepsis using nationwide real-world data from Japan.

**Methods:**

This retrospective cohort study used nationwide Japanese data from a medical reimbursement system database. Patients aged ≥20 years with both presumed infections and acute organ dysfunction who were admitted to hospitals from the outpatient department or emergency department between 2010 and 2017 were enrolled. We described the initial choices of antimicrobials for patients with sepsis stratified by intensive care unit (ICU) or ward.

**Results:**

There were 1,195,741 patients with community-onset sepsis; of these, 1,068,719 and 127,022 patients were admitted to the wards and ICU, respectively. Third-generation cephalosporins and carbapenem were most commonly used for patients with community-onset sepsis. We found that 1.7% and 6.0% of patients initially used antimicrobials for methicillin-resistant *Staphylococcus aureus* coverage in the wards and ICU, respectively. Although half of the patients initially used antipseudomonal agents, only a few patients used a combination of antipseudomonal agents. Moreover, few patients initially used a combination of antimicrobials to treat methicillin-resistant *Staphylococcus aureus* and *Pseudomonas* sp.

**Conclusion:**

Third-generation cephalosporins and carbapenem were most frequently used for patients with sepsis. A combination therapy of antimicrobials for drug-resistant bacteria coverage was rarely provided to these patients.

## Highlights

• Third-generation cephalosporins were the most frequently used for sepsis in wards.
• Carbapenems were the most frequently used for sepsis in intensive care units in Japan.
• Half of the patients with sepsis initially used an antipseudomonal microbial.
• A combination therapy of antimicrobials for drug-resistant bacteria was rare.

## Background

Sepsis is commonly encountered in healthcare facilities [1]. It is a life-threatening syndrome that occasionally requires intensive care. To reduce mortality among patients with sepsis, it is necessary to initiate timely, appropriate, empiric, broad-spectrum antimicrobial administration. Inappropriate initial antimicrobial use is associated with high mortality [2]. In fact, the most challenging task is to empirically select an appropriate antimicrobial agent without over- or under-diagnosis.

The sepsis campaign guidelines recommend the early administration of appropriate broad-spectrum antimicrobials and the use of methicillin-resistant *Staphylococcus aureus* (MRSA)-covering antibiotics or a combination therapy against multi-drug-resistant (MDR) pathogens only in suspected MRSA or MDR bacterial infection cases [1]. This forms a part of the practice for infectious disease treatment, and the guidelines do not provide specific guidance. However, clinicians in the emergency department (ED) should be able to select the most appropriate antimicrobials that could be used immediately while waiting for detailed information about the infection.

Few reports have investigated the empirical antimicrobial agents used by clinicians for community-onset sepsis. Without a clear guideline and evidence for the selection of appropriate antimicrobials, many clinicians make decisions based on their experience while managing these patients. Therefore, we aimed to describe the empiric antimicrobial of choice for patients with community-onset sepsis using nationwide real-world data in Japan.

## Methods

### Study cohort

This retrospective cohort study used nationwide Japanese data obtained from the medical reimbursement system for acute care “Diagnosis Procedure Combination (DPC).” This system included 1237 acute care hospitals, accounting for 71.5% of the Japanese acute care hospitals in 2017 [3,4].

The study participants were aged ≥20 years, had both presumed infections and acute organ dysfunction and were admitted to hospitals between 2010 and 2017 [4]. There were no exclusion criteria. However, we only chose patients with community-onset sepsis because the pathogens of community-acquired and healthcare-associated infections are quite different. Community-onset sepsis was diagnosed when both blood culture collection and new antimicrobial administration were performed within 48 hours of admission. In cases with multiple hospitalizations, we considered only first hospitalization.

### Data collection and definitions

The definition of sepsis (severe infections and acute organ dysfunction) was the same as that in our previous report [4]. Presumed infections were defined as blood culture collection and new antimicrobial administration for >4 days. Intravenous antimicrobial administration was required within 2 calendar days before or after blood culture collection. Antimicrobials were administered for ≥4 days. Although antimicrobials must have been administered for <4 days if patients died in <4 days, those were included. The used database excluded laboratory data. Therefore, we defined acute organ dysfunction using diagnostic codes and organ-specific treatment data.

Diagnostic codes were assigned based on primary diagnosis on admission, comorbidities on admission, and posthospitalization complications and were defined using the international statistical classification of diseases and related health problems, 10th revision (ICD-10) in this DPC database (Table S1). The focus of infection was also drawn by referring to ICD-10 codes for presumed foci of infection (Table S2). Information regarding acute organ dysfunction was obtained using diagnostic data from the ICD-10 codes for hepatic dysfunction, thrombocytopenia/coagulopathy, and acidosis (Table S3).

In cases where combination therapy excluded drug-resistant pathogens such as MRSA or *Pseudomonas species*, monotherapy was defined as the administration of any single antimicrobial, and combination therapy was defined as the concomitant administration of two or more antimicrobials of different mechanistic classes. The use of a combination of antimicrobials from the same mechanistic class was not classified as combination therapy. Typical combination therapy includes two cell-wall-active agents (β-lactam or glycopeptide) and aminoglycoside, fluoroquinolone, or macrolide/clindamycin [5]. Antimicrobials for *Pseudomonas species* infections include aminoglycosides, carbapenems, antipseudomonal cephalosporins, fluoroquinolone, antipseudomonal penicillin plus beta-lactamase inhibitors, monobactams, phosphonic acids, and polymyxins. The antipseudomonal combination was defined as two or more antipseudomonal agents that were concomitantly administered. The combination included both beta-lactam and non-beta-lactam antipseudomonal antimicrobial agents (aminoglycoside, quinolone, and other types of antipseudomonal agents such as fosfomycin, colistin, and aztreonum). Data regarding the use of vasopressors, renal replacement therapy, and ventilators were extracted for use during hospitalization.

As outcomes, we collected data for in-hospital mortality, length of antibiotic treatment, and duration of hospital and ICU stay.

### Analysis

Descriptive statistics included percentages for categorical variables and the median (interquartile range [IQR]) for continuous variables. We also stratified patients with sepsis into ICU or ward patients. We also described year-to-year changes in antimicrobial use trends.

For subgroup analyses, we separately analyzed patients with sepsis having four major infection sites, including community-acquired pneumonia (CAP), urogenital infection, abdominal infection, and bone and soft tissue infection. We performed statistical analyses using R software (Version 3.6.2).

## Results

We extracted the data of 1,451,483 patients with sepsis from the medical reimbursement system for acute care. Of these, 1,195,741 (82.4%) patients were admitted from the outpatient or ED. Overall, 1,068,719 (89.4%) patients were admitted to a ward, and 127,022 (10.6%) patients were admitted to ICU.

Table 1 shows the baseline characteristics of the study participants. The median age of the participants was 76 years (IQR: 62-84 years), and 55.9% were men. The most common comorbidity was hypertension (23.3%), followed by malignancies (22.5%) and diabetes (19.0%). The most common site of infection was the lung (38.0%), followed by the abdomen (15.7%), urinary tract (7.8%), and bone and soft tissues (5.3%). The proportions of patients who used ventilators in the ward and ICU were 9.8% and 51.7%, respectively.

**Table 1:** The baseline characteristics of the study participants.

		Overall	Ward	Intensive care unit
		1,195,741	1,068,719	127,022
Age, year, median [interquartile range]		76 [62-84]	76.00 [62-84]	73.00 [61-81]
Male, n (%)		668,582 (55.9)	591,379 (55.3)	77,203 (60.8)
Comorbidity	Malignant tumor, n (%)	268,914 (22.5)	244,474 (22.9)	24,440 (19.2)
	Hypertension, n (%)	278,775 (23.3)	245,120 (22.9)	33,655 (26.5)
	Diabetes mellitus, n (%)	227,672 (19.0)	199,016 (18.6)	28,656 (22.6)
	Heart failure, n (%)	191,537 (16.0)	160,019 (15.0)	31,518 (24.8)
	Cerebrovascular disease, n (%)	142,754 (11.9)	125,094 (11.7)	17,660 (13.9)
	Ischemic heart disease, n (%)	99,365 (8.3)	79,654 (7.5)	19,711 (15.5)
	Chronic respiratory disease, n (%)	148,754 (12.4)	138,162 (12.9)	10,592 (8.3)
	Chronic renal failure, n (%)	36,708 (3.1)	30,977 (2.9)	5,731 (4.5)
Focus of infection	Respiratory, n (%)^a^	454,313 (38.0)	418,429 (39.2)	35,884 (28.3)
	Urogenital, n (%)	93,428 (7.8)	87,056 (8.1)	6,372 (5.0)
	Abdominal, n (%)	187,639 (15.7)	162,013 (15.2)	25,626 (20.2)
	Bone and soft tissue, n (%)	63163 (5.3)	56084 (5.2)	7079 (5.6)
	Meninges/brain/spinal cord, n (%)	28,788 (2.4)	24,196 (2.3)	4,592 (3.6)
	Heart, n (%)	42,882 (3.6)	37,931 (3.5)	4,951 (3.9)
	Sexually transmitted disease, n (%)	13,060 (1.1)	11,909 (1.1)	1,151 (0.9)
	Blood, n (%)	1,706 (0.1)	1,596 (0.1)	110 (0.1)
	Unknown, n (%)	152,418 (12.7)	134,212 (12.6)	18,206 (14.3)
Vasopressor use		109,552 (9.2)	82,002 (7.7)	27,550 (21.7)
Renal replacement therapy use		39,146 (3.3)	20,444 (1.9)	18,702 (14.7)
Ventilator use		169,917 (14.2)	104,295 (9.8)	65,622 (51.7)

a Respiratory included pneumonia and pyothorax.

**Figure 1:**
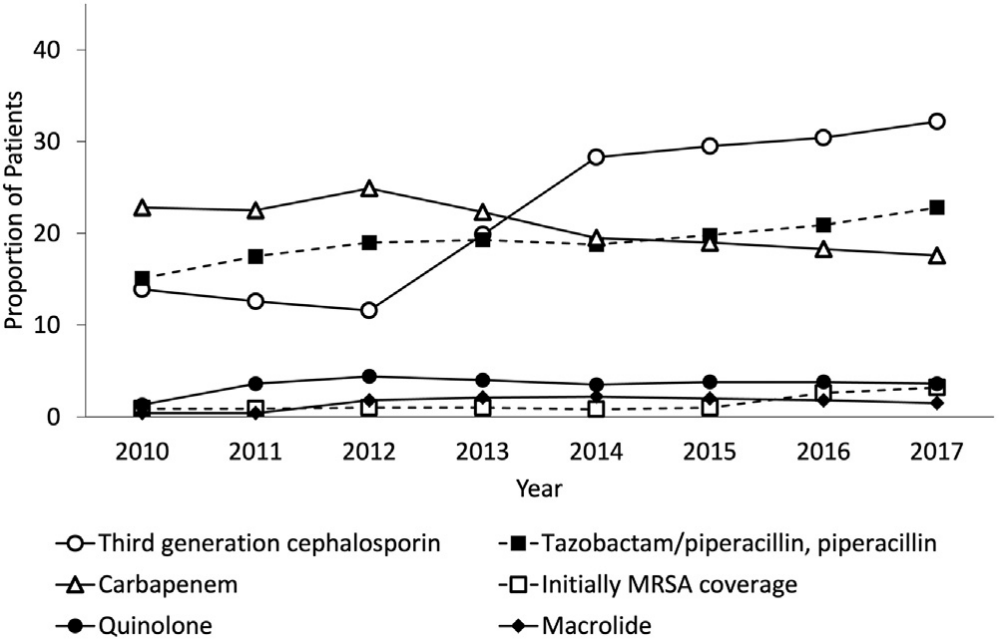
Annual change of initial empiric antimicrobial choice in the ward. MRSA, methicillin-resistant *Staphylococcus aureus*.

**Figure 2:**
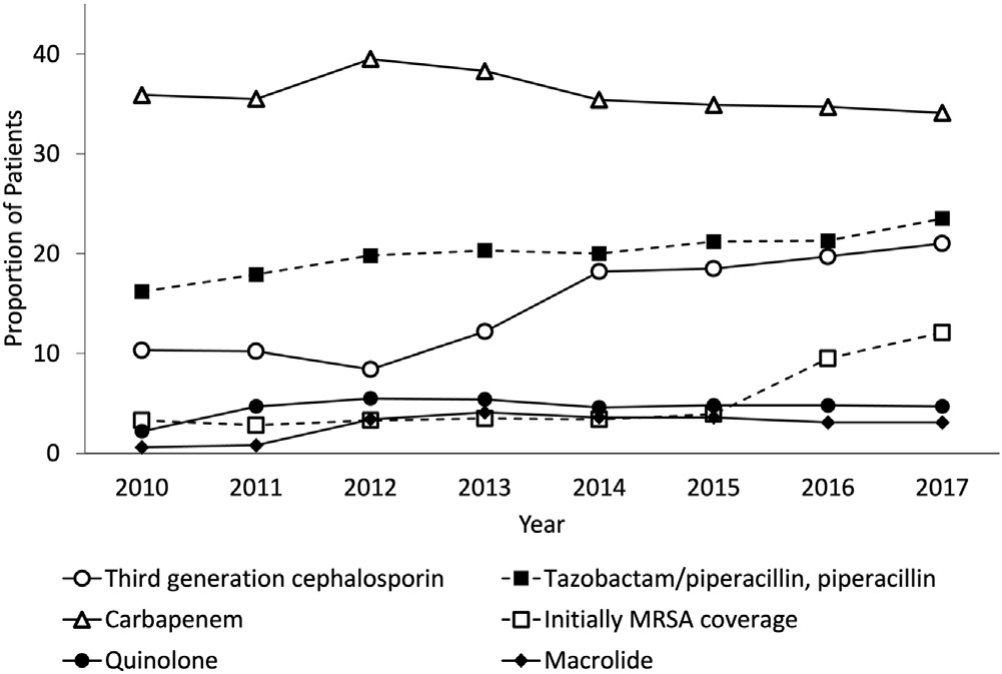
Annual change of initial empiric antimicrobial choice in the intensive care unit. MRSA, methicillin-resistant *Staphylococcus aureus*.

Table 2 shows the initial empiric antimicrobial of choice among patients with community-onset sepsis. Overall, third-generation cephalosporins were most commonly used (24.3%), followed by carbapenem (21.7%) and tazobactam/piperacillin (19.9%). In the ICU, carbapenem was most commonly used (35.8%), followed by tazobactam/piperacillin (20.7%) and third-generation cephalosporins (16.4%). Overall, 7.7% of the patients received combination therapy that excluded drug-resistant pathogens. Furthermore, 1.7% and 6.0% of the patients initially used antimicrobials for MRSA coverage in the ward and ICU, respectively. Although half of the patients initially used antipseudomonal microbial monotherapy (overall: 47.1, ward: 46.1%, and ICU: 55.4%), only a few patients used antipseudomonal combinations (overall: 3.7%, ward: 3.4%, and ICU: 6.3%). Moreover, a small proportion of patients initially used antimicrobial combinations to cover MRSA and *Pseudomonas species* infections (overall: 1.5%, ward: 1.1%, and ICU: 4.7%). Figure 1, Figure 2 show the annual change in initial empiric antimicrobial choice in the ward and ICU. The frequency of third-generation cephalosporin and carbapenem use in the ward switched from 2014, although the annual change of frequency of antimicrobials in the ICU did not clinically change.

**Table 2:** The initial empiric antimicrobials of choice among patients with community-onset sepsis.

		Overall	Ward	Intensive care unit
Antibiotics, n (%)		1,195,741	1,068,719	127,022
Penicillin derivative		83,357 (7.0)	79,443 (7.4)	3914 (3.1)
Ampicillin/sulbactam		76,878 (6.4)	67,718 (6.3)	9160 (7.2)
Tazobactam/piperacillin, piperacillin		238,533 (19.9)	212,205 (19.9)	26,328 (20.7)
Sulbactam/cefoperazone		12,448 (1.0)	11,661 (1.1)	787 (0.6)
First-generation cephalosporin		68,487 (5.7)	50,653 (4.7)	17,834 (14.0)
Second-generation cephalosporin		183,743 (15.4)	166,101 (15.5)	17,642 (13.9)
Third-generation cephalosporin		291,156 (24.3)	270,342 (25.3)	20,814 (16.4)
Third-generation cephalosporin against pseudomonas		14,903 (1.2)	14,044 (1.3)	859 (0.7)
Fourth-generation cephalosporin against pseudomonas		57,240 (4.8)	53,797 (5.0)	3443 (2.7)
Carbapenem		259,305 (21.7)	213,864 (20.0)	45,441 (35.8)
Aminoglycoside		35,986 (3.0)	32,014 (3.0)	3972 (3.1)
Quinolone		45,123 (3.8)	39,076 (3.7)	6047 (4.8)
Tetracycline		16,617 (1.4)	15,187 (1.4)	1430 (1.1)
Macrolide		22,092 (1.8)	18,113 (1.7)	3979 (3.1)
Metronidazole		2425 (0.2)	1,850 (0.2)	575 (0.5)
Clindamycin		24,217 (2.0)	20,343 (1.9)	3874 (3.0)
Vancomycin		16,762 (1.4)	11,723 (1.1)	5039 (4.0)
Other anti-MRSA drugs		8967 (0.7)	6282 (0.6)	2685 (2.1)
Others^a^		4694 (0.4)	3930 (0.4)	764 (0.6)
Antimicrobials combination	Mono therapy	954,300 (79.8)	866,968 (81.1)	87,332 (68.8)
	Combination therapy without drug-resistant pathogens	92,335 (7.7)	77,779 (7.3)	14,556 (11.5)
Initially MRSA coverage		25,458 (2.1)	17,845 (1.7)	7613 (6.0)
Initially pseudomonas coverage	Mono therapy	608,233 (50.9)	78,266 (61.6)	529,967 (49.6)
	Antipseudomonal combination	26,505 (2.2)	21,499 (2.0)	5006 (3.9)
Initially MRSA & pseudomonas coverage		18,117 (1.5)	12,154 (1.1)	5963 (4.7)

MRSA, methicillin-resistant *Staphylococcus aureus.*

a Included Aztreonam, Fosfomycin, Quinupristin / Dalfopristin, Chloramphenicol succinate, Trimethoprim-sulfamethoxazole, Colistin Sodium Methanesulfonate, and Methenamine.

The overall in-hospital mortality rate was 12.7% (Table 3). The mortality rates for patients in the ward and ICU were 12.4% and 15.7%, respectively. Table S4 shows the in-hospital mortalities based on different selections of initial empiric antimicrobials. Patients with antipseudomonal combination or initial MRSA and *Pseudomonas* coverage had higher in-hospital mortality than other patients. Antimicrobials were used for 9 (7-15) and 12 (8-20) days in the ward and ICU before discharge from the hospital, respectively. Among ICU patients, the median length of ICU stay was 4 days (IQR: 2-8 days). The median length of hospital stay among patients in the wards and ICU was 16 (10-30) and 25 (15-42) days, respectively.

**Table 3:** Outcomes among patients with community-onset sepsis.

	Overall	Ward	ICU
	1,195,741	1,068,719	1,27,022
Length of antibiotic treatment, days, median [IQR]	10 [7-15]	9 [7-15]	12 [8-20]
Length of hospital stay, days, median [IQR]	17 [10-31]	16 [10-30]	25 [15-42]
Length of ICU stay, days, median [IQR]	0 [0-0]	NA	4 [2-8]
In-hospital mortality, n (%)	152,245 (12.7)	132,280 (12.4)	19,965 (15.7)

ICU, intensive care unit; IQR, interquartile range; NA, not available.

In the subgroup analysis (Table S5-1), 448,567 septic patients had CAP, and 6.1%, 3.7%, and 2.4% of patients used quinolones, macrolides, and tetracycline, respectively. Thus, 11.9% of the overall patients, 11.2% of the ward patients, and 19.8% of the ICU patients were initially covered for atypical pneumonia. In the ICU, the coverage for MRSA and *Pseudomonas species* was 5.4% and 64.0%, respectively.

## Discussion

Our nationwide study showed that third-generation cephalosporins were most frequently used as initial antimicrobial agents for community-onset sepsis in Japan. In the ICU, carbapenem was the most commonly used antimicrobial. Although half of the patients with community-onset sepsis were initially administered antipseudomonal agents, only some patients used antimicrobials for MRSA coverage. In the ED, patients with sepsis were rarely administered a combination of antipseudomonal drugs or a combination of antimicrobials for drug-resistant pathogens, such as MRSA and *Pseudomonas* sp.

Early administration of antimicrobials is recommended for patients with sepsis because it is one of the most effective interventions to reduce mortality [1]. Ideally, they should be administered within 1 hour from recognition of sepsis; therefore, the time for selecting appropriate antimicrobials is limited. Generally, *Pseudomonas* sp. infections are not routinely considered community-onset infections, with few exceptions, because they are often caused by different organisms, unlike in the case of healthcare-associated infections [6]. Study participants experienced sepsis, and the severity of the disease should have been high compared with those just with an infection, but antipseudomonal drugs may be overused.

Unfortunately, the concordance between bacterial susceptibility and antimicrobials could not be determined in this study because there was no data on drug-resistant bacteria in patients with sepsis in Japan. Broad-spectrum antimicrobials, especially those covering drug-resistant pathogens, administered in our results would be overboard [7]. Notably, after 2014, third-generation cephalosporins were more commonly used than carbapenems in wards; however, no change was observed in ICU. This trend probably indicates the beginning of a response to prevent antimicrobial resistance and promote the appropriateness of antimicrobial choice in Japan. The empiric regimen can be determined by assessing the risks from patient and epidemiological aspects, such as signs, symptoms, severity of illness, and suspected site of infection [8]. The safety of early aggressive and appropriate antimicrobials and the possible adverse effects associated with unnecessary treatment with antimicrobials should be assessed in the future [9].

Our results show that antimicrobial usage for MRSA coverage was uncommon for patients with community-onset sepsis in Japan. Empiric use of antimicrobials for MRSA coverage varies among patients with sepsis [1]. The prevalence of MRSA infection among patients with sepsis might be decreasing [10] and varies worldwide [7]. In our study, the mortality rate was not higher than that reported in previous studies [11]. Failure to cover MRSA in a patient with MRSA-induced sepsis may be harmful, but the reverse is also true. Further studies are warranted to facilitate the rapid diagnosis of MRSA infection because clinicians still rely on vague clinical decisions.

Regarding the use of combination therapies, previous studies have not yet proven the effectiveness of combination therapy for MDR pathogens [12,13]. Therefore, the guideline recommends that two or more antimicrobials with gram‐negative coverage should be used only for patients with sepsis and those at high risk for MDR pathogen infection. Our study showed that combination therapy based on antipseudomonal drugs was not commonly used in Japan. Additionally, combination therapy for both MRSA and *Pseudomonas* sp. coverage is also rare; however, it may be used for highly severe or complicated cases and in patients in whom the site of infection is difficult to diagnose. Another combination therapy involved no coverage of drug-resistant pathogens. A typical example of coverage is CAP, which empirically covers both typical and atypical pneumonia. Therefore, it includes a combination of nonantipseudomonal β-lactams and any respiratory quinolones, tetracycline, or macrolides. In the CAP subgroup, approximately 20% and 11% of patients were covered by these combination therapies in ICUs and wards, respectively. These therapies accounted for half of the antimicrobial combination therapies that did not cover drug-resistant pathogens among all participants. This coverage rate of atypical pneumonia was higher than that in a previous study [14], although their setting was different from our study. Limited data are available for the superiority of antimicrobial monotherapy over combination therapy in the treatment of CAP [15]. However, it is recommended that patients with comorbidities, especially those in the ICU, should receive broader-spectrum treatment that includes not only typical or atypical pneumonia but also *Pseudomonas* sp. infections.

Regarding the duration of antibiotic treatment, patients in the ICU used antibiotics for a longer duration than those in the ward. However, these data were acquired from acute care hospitals. The duration of antibiotic therapy for patients in ICU was defined as the time from when they were transferred from the ICU to a ward until they were discharged. The duration of antibiotic therapy for patients in the ward was defined as the time from admission to the ward until they were discharged. Regarding the outcomes, the mortality rates were slightly lower in the present study than those in previous studies [1,[16], [17], [18]. Patients with mild sepsis could have been included more than those from the data collected from clinical practice because of data from the medical reimbursement system. The same trend was observed in a previous study that used data extracted from electronic medical records [19].

### Limitation

First, our study was conducted only in Japan. The health economic system influences the choice of medications in each country. Because Japan has universal health insurance, decisions could be easily made based on the medicine requirement and not on economics as compared with other countries. Second, we did not have any information on pathogenic bacteria. Our results of antibiotic appropriateness were presumed based on past literature and antimicrobial agents. Some patients might not have received appropriate antimicrobials [20]. Third, the percentage of causative infections leading to sepsis was slightly different from that reported in other studies [21], [22], [23] because of different study settings, disease classification, and inclusion and exclusion criteria of the participants. Fourth, we might have included some patients with healthcare-associated infection in our study population because we only retrospectively selected patients with sepsis diagnosed within 48 hours of admission as community-onset based on real-world data. Fifth, because we did not have laboratory data, we may have either underestimated or overestimated the patient's selection as a sepsis definition. However, we presumably captured most patients with sepsis and severe infection in the DPC data. Finally, we did not have time data for antimicrobial administration. Unmeasured quality of medical care may have affected the results of this study. It will be necessary to assess the quality of care in future research on antimicrobials and the prognosis of patients with sepsis.

## Conclusion

Our nationwide study showed that third-generation cephalosporins and carbapenem were the most frequently used initial antimicrobials in the ward and ICU, respectively, among patients with community-onset sepsis in Japan. Although half of the patients with community-onset sepsis were initially administered antipseudomonal agents, only some patients were administered antimicrobials for MRSA coverage. It was rare for patients with community-onset sepsis to be administered a combination therapy for drug-resistant bacteria coverage.

## Declarations of competing interest

The authors have no competing interests to declare.

## Appendix Supplementary materials

Image, application 1

Image, application 2
