# Supplementary material for: Epidemiology and patterns of empiric antimicrobial therapy practice in patients with community-onset sepsis using data from a Japanese nationwide medical claims database—the Japan Sepsis Alliance (JaSA) study group
Source: IJID Reg. 2024 Jan 3;10:162–7. doi: 10.1016/j.ijregi.2024.01.002 (PMC10835350; doi:10.1016/j.ijregi.2024.01.002)
Supplement: Supplementary file 1 [file mmc1.docx]

Epidemiology and patterns of empiric antimicrobial therapy practice in patients with community-onset sepsis using data from a Japanese nationwide medical claims database — the Japan Sepsis Alliance (JaSA) study group

Toshikazu Abe; Iriyama Hiroki; Taro Imaeda; Akira Komori; Takehiko Oami; Tuerxun Aizimu; Nozomi Takahashi; Yasuo Yamao; Satoshi Nakagawa; Hiroshi Ogura; Yutaka Umemura; Asako Matsushima; Kiyohide Fushimi; Nobuaki Shime; Taka-aki Nakada

***Online data Supplement***

**Table S1.** Comorbidity categories with corresponding ICD-10

| Comorbidity | ICD-10 codes |
| --- | --- |
| Malignant tumor | C00-C97, D00-D09 |
| Hypertension | I10-I15 |
| Diabetes mellitus | E10-E14 |
| Heart failure | I50 |
| Cerebrovascular disease | I60-I69 |
| Ischemic heart disease | I20-I25 |
| Chronic respiratory disease | J40-J47 |
| Chronic renal failure | N18 |

**Table S2.** Focus of infection with corresponding ICD-10

| Focus of infection | ICD-10 codes |
| --- | --- |
| Respiratory | A15-A16, J00-J06, J09-J18, J20-J22, J31-J32, J35-J37, J39.0, J39.1, J85-J86 |
| Urogenital | A18.1, A51.0, A54.0-A54.2, A56.0-A56.2, A59.0, A60.0, N30.0, N30.8, N39.0, N41.0-N41.3, N45, N49.0-N49.2, N70-N77, O23 |
| Abdominal | A00-A09, A18.3, A42.1, A74.8, K35-K38, K57.0, K57.2, K57.4, K57.8, K61, K63.0, K63.1, K65, K67, K75.0, K80.0, K80.1, K80.3, K80.4, K81, K83.0 |
| Bone and soft tissue | A18.0, A18.4, A26.0, A28.1, A31.1, A31.8, A32.0, A36.3, A42.2, A43.1, A46, A48.0, L00-L08, M00, M01.0, M46.3, M46.5, M49.1-M49.3, M60.0, M86.0, M86.1, M86.65, M86.66, M86.69, M86.99 |
| Blood | A19 (Miliary tuberculosis), A40.0 (Invasive group A streptococcal disease), A49.0 (Methicillin susceptible Staphylococcus aureus bacteremia), A49.1 (Invasive pneumococcal disease), A49.9 (bacteremia) |

**Table S3.** Acute organ dysfunction categories with corresponding ICD-10

| Organ dysfunction | ICD-10 codes |
| --- | --- |
| Renal | N00.9（Acute nephritis syndrome） |
|  | N10（Acute tubulointerstitial nephritis） |
|  | N17.0（Shock kidney, Acute parenchymal renal failure, Acute kidney tubular necrosis） |
|  | N17.1（Acute renal cortical necrosis） |
|  | N17.8（Acute prerenal failure） |
|  | N17.9（Acute kidney injury） |
| Hepatic | K72.0（Acute liver failure） |
|  | K72.9（Liver failure, details unknown） |
|  | K76.8（Shock liver） |
| Thrombocytopenia | D69.5（Secondary thrombocytopenia） |
|  | D69.6（Thrombocytopenia） |
| Coagulopathy | D65（Disseminated intravascular coagulation） |
|  | D68.9（Other and unspecified coagulation disorders） |
| Acidosis | E87.2（Acidosis, metabolic or lactic） |
