# Supplementary material for: Epidemiology and patterns of empiric antimicrobial therapy practice in patients with community-onset sepsis using data from a Japanese nationwide medical claims database—the Japan Sepsis Alliance (JaSA) study group
Source: IJID Reg. 2024 Jan 3;10:162–7. doi: 10.1016/j.ijregi.2024.01.002 (PMC10835350; doi:10.1016/j.ijregi.2024.01.002)
Supplement: Supplementary file 2 [file mmc2.docx]

Epidemiology and patterns of empiric antimicrobial therapy practice in patients with community-onset sepsis using data from a Japanese nationwide medical claims database — the Japan Sepsis Alliance (JaSA) study group

Toshikazu Abe; Iriyama Hiroki; Taro Imaeda; Akira Komori; Takehiko Oami; Tuerxun Aizimu; Nozomi Takahashi; Yasuo Yamao; Satoshi Nakagawa; Hiroshi Ogura; Yutaka Umemura; Asako Matsushima; Kiyohide Fushimi; Nobuaki Shime; Taka-aki Nakada

***Online data Supplement 2***

Table S4: in-hospital mortalities based on the different selections of initial empiric antimicrobials.

|  |  | Overall | Ward | ICU |
| --- | --- | --- | --- | --- |
| Antibiotics, n (%) |  | 1,195,741 | 1,068,719 | 127,022 |
| Penicillin derivative |  | 2151 (2.6) | 1745 (2.2) | 406 (10.4) |
| Ampicillin/sulbactam |  | 10175 (13.2) | 9034 (13.3) | 1141 (12.5) |
| Tazobactam/piperacillin, piperacillin |  | 40045 (16.8) | 35053 (16.5) | 4992 (19.0) |
| Sulbactam/cefoperazone |  | 1518 (12.2) | 1425 (12.2) | 93 (11.8) |
| First generation cephalosporin |  | 6474 (9.5) | 4409 (8.7) | 2065 (11.6) |
| Second generation cephalosporin |  | 12725 (6.9) | 10982 (6.6) | 1743 (9.9) |
| Third generation cephalosporin |  | 30840 (10.6) | 27820 (10.3) | 3020 (14.5) |
| Third generation cephalosporin against pseudomonas |  | 1812 (12.2) | 1650 (11.7) | 162 (18.9) |
| Fourth generation cephalosporin against pseudomonas |  | 11603 (20.3) | 10776 (20.0) | 827 (24.0) |
| Carbapenem |  | 45535 (17.6) | 37223 (17.4) | 8312 (18.3) |
| Aminoglycoside |  | 2474 (6.9) | 1978 (6.2) | 496 (12.5) |
| Quinolone |  | 8554 (19.0) | 7039 (18.0) | 1515 (25.1) |
| Tetracycline |  | 2785 (16.8) | 2442 (16.1) | 343 (24.0) |
| Macrolide |  | 3983 (18.0) | 3043 (16.8) | 940 (23.6) |
| Metronidazole |  | 424 (17.5) | 312 (16.9) | 112 (19.5) |
| Clindamycin |  | 3574 (14.8) | 3009 (14.8) | 565 (14.6) |
| Vancomycin |  | 3301 (19.7) | 2250 (19.2) | 1051 (20.9) |
| Other anti-MRSA drugs |  | 2099 (23.4) | 1507 (24.0) | 592 (22.0) |
| Others† |  | 933 (19.9) | 717 (18.2) | 216 (28.3) |
| Antimicrobials combination | Mono therapy | 118,411 (12.4) | 105,439 (12.2) | 12,972 (14.9) |
|  | Combination therapy without drug-resistant pathogens | 13,456 (14.6) | 10,610 (13.6) | 2,846 (19.6) |
| Initially MRSA coverage |  | 5349 (21.0) | 3730 (20.9) | 1619 (21.3) |
| Initially pseudomonas coverage | Mono therapy | 94799 (16.3) | 81632 (16.1) | 13167 (18.0) |
|  | Antipseudomonal combination | 5677 (21.4) | 4448 (20.7) | 1229 (24.6) |
| Initially MRSA & pseudomonas coverage |  | 4275 (23.6) | 2888 (23.8) | 1387 (23.3) |
| ICU: intensive care unit, MRSA: methicillin-resistant *Staphylococcus aureus*  †included Aztreonam, Fosfomycin, Quinupristin / Dalfopristin, Chloramphenicol succinate, Trimethoprim sulfamethoxazole, Colistin Sodium Methanesulfonate, Methenamine | | | | |

Table S5-1. The initial empiric antimicrobials of choice and outcomes among septic patients with community-acquired pneumonia

|  |  | Overall | Ward | ICU |
| --- | --- | --- | --- | --- |
| Antibiotics, n (%) |  | 448,567 | 413,521 | 35,046 |
| Age, year, median [IQR] |  | 76 [63–84] | 77 [63–85] | 74 [62–82] |
| Male, n (%) |  | 271,109 (60.4) | 248,312 (60.0) | 22,797 (65.0) |
| Comorbidity | Malignant tumor, n (%) | 88,350 (19.7) | 82,725 (20.0) | 5,625 (16.1) |
|  | Hypertension, n (%) | 103,235 (23.0) | 94,295 (22.8) | 8,940 (25.5) |
|  | Diabetes mellitus, n (%) | 82,093 (18.3) | 74,117 (17.9) | 7,976 (22.8) |
|  | Heart failure, n (%) | 90,026 (20.1) | 78,608 (19.0) | 11,418 (32.6) |
|  | Cerebrovascular disease, n (%) | 48,751 (10.9) | 44,176 (10.7) | 4,575 (13.1) |
|  | Ischemic heart disease, n (%) | 37,008 (8.3) | 31,590 (7.6) | 5,418 (15.5) |
|  | Chronic respiratory disease, n (%) | 99,931 (22.3) | 94,344 (22.8) | 5,587 (15.9) |
|  | Chronic renal failure, n (%) | 13,610 (3.0) | 11,967 (2.9) | 1,643 (4.7) |
| Vasopressor use |  | 30,309 (6.8) | 23,360 (5.6) | 6,949 (19.8) |
| RRT use |  | 9,717 (2.2) | 5,669 (1.4) | 4,048 (11.6) |
| Ventilator use |  | 60,236 (13.4) | 38,967 (9.4) | 21,269 (60.7) |
| Penicillin derivative |  | 34442 (7.7) | 33222 (8.0) | 1220 (3.5) |
| Ampicillin/sulbactam |  | 37,284 (8.3) | 34,201 (8.3) | 3,083 (8.8) |
| Tazobactam/piperacillin, piperacillin |  | 101,114 (22.5) | 92,146 (22.3) | 8,968 (25.6) |
| Sulbactam/cefoperazone |  | 1,098 (0.2) | 1,020 (0.2) | 78 (0.2) |
| First generation cephalosporin |  | 9,509 (2.1) | 7,417 (1.8) | 2,092 (6.0) |
| Second generation cephalosporin |  | 45,413 (10.1) | 42,611 (10.3) | 2,802 (8.0) |
| Third generation cephalosporin |  | 127,714 (28.5) | 120,083 (29.0) | 7,631 (21.8) |
| Third generation cephalosporin against psedomonas |  | 4,908 (1.1) | 4,655 (1.1) | 253 (0.7) |
| Fourth generation cephalosporin against psedomonas |  | 21,921 (4.9) | 20,840 (5.0) | 1,081 (3.1) |
| Carbapenem |  | 80,723 (18.0) | 69,278 (16.8) | 11,445 (32.7) |
| Aminoglycoside |  | 41,56 (0.9) | 3,615 (0.9) | 541 (1.5) |
| Quinolone |  | 27,261 (6.1) | 23,605 (5.7) | 3,656 (10.4) |
| Tetracycline |  | 10,775 (2.4) | 9,977 (2.4) | 798 (2.3) |
| Macrolide |  | 16,511 (3.7) | 13,732 (3.3) | 2,779 (7.9) |
| Metronidazole |  | 582 (0.1) | 470 (0.1) | 112 (0.3) |
| Clindamycin |  | 8,164 (1.8) | 7,292 (1.8) | 872 (2.5) |
| Vancomycin |  | 5,068 (1.1) | 3,806 (0.9) | 1,262 (3.6) |
| Other anti–MRSA drugs |  | 2,561 (0.6) | 1,907 (0.5) | 654 (1.9) |
| Others |  | 1,501 (0.3) | 1,179 (0.3) | 322 (0.9) |
| Antimicrobials combination | Mono therapy | 366,017 (81.6) | 343,036 (83.0) | 22,981 (65.6) |
|  | Combination therapy without drug-resistant pathogens | 37,707 (8.4) | 3,1382 (7.6) | 6,325 (18.0) |
| Atypical pneumonia coverage | | 53,181 (11.9) | 46,234 (11.2) | 6,947 (19.8) |
| Initially MRSA coverage |  | 7,573 (1.7) | 5,679 (1.4) | 1,894 (5.4) |
| Initially pseudomonas coverage | Mono therapy | 200,184 (44.6) | 181,150 (43.8) | 19,034 (54.3) |
|  | Antipseudomonal combination | 19,815 (4.4) | 16,412 (4.0) | 3,403 (9.7) |
| Initially MRSA & pseudomonas coverage |  | 5,752 (1.3) | 4,188 (1.0) | 1,564 (4.5) |
| Length of antibiotic treatment, days, median [IQR] |  | 9 [6–13] | 8 [6–13] | 10 [7–16] |
| Length of hospital stay, days, median [IQR] |  | 15 [9–28] | 15 [9–27] | 22 [13–39] |
| Length of ICU stay, days, median [IQR] |  | 0 [0–0] | NA | 5 [2–9] |
| In-hospital mortality, n (%) |  | 61,251 (13.7) | 54,043 (13.1) | 7,208 (20.6) |
| Atypical pneumonia coverage included quinolone, tetracycline, and macrolide | | | | |
| ICU: intensive care unit, IQR: interquartile range, NA: not available, MRSA: methicillin-resistant *Staphylococcus aureus* | | | | |

Table S5-2. The initial empiric antimicrobials of choice and outcomes among septic patients with urinary tract infection

|  |  | Overall | Ward | ICU |
| --- | --- | --- | --- | --- |
| Antibiotics, n (%) |  | 93,428 | 87,056 | 6,372 |
| Age, year, median [IQR] |  | 81 [72–88] | 82 [72–88] | 79 [69–85] |
| Male, n (%) |  | 54,969 (58.8) | 51,361 (59.0) | 3,608 (56.6) |
| Comorbidity | Malignant tumor, n (%) | 14,709 (15.7) | 13,823 (15.9) | 886 (13.9) |
|  | Hypertension, n (%) | 22,379 (24.0) | 20,877 (24.0) | 1,502 (23.6) |
|  | Diabetes mellitus, n (%) | 17,726 (19.0) | 16,261 (18.7) | 1,465 (23.0) |
|  | Heart failure, n (%) | 14,838 (15.9) | 13,557 (15.6) | 1,281 (20.1) |
|  | Cerebrovascular disease, n (%) | 15,718 (16.8) | 14,477 (16.6) | 1,241 (19.5) |
|  | Ischemic heart disease, n (%) | 6,789 (7.3) | 6,086 (7.0) | 703 (11.0) |
|  | Chronic respiratory disease, n (%) | 59,63 (6.4) | 5,606 (6.4) | 357 (5.6) |
|  | Chronic renal failure, n (%) | 2,969 (3.2) | 2,708 (3.1) | 261 (4.1) |
| Vasopressor use |  | 47,87 (5.1) | 3,815 (4.4) | 972 (15.3) |
| RRT use |  | 1,589 (1.7) | 940 (1.1) | 649 (10.2) |
| Ventilator use |  | 5,925 (6.3) | 3,755 (4.3) | 2,170 (34.1) |
| Penicillin derivative |  | 3,122 (3.3) | 2,971 (3.4) | 151 (2.4) |
| Ampicillin/sulbactam |  | 3,817 (4.1) | 3,530 (4.1) | 287 (4.5) |
| Tazobactam/piperacillin, piperacillin |  | 18,398 (19.7) | 16,886 (19.4) | 1,512 (23.7) |
| Sulbactam/cefoperazone |  | 352 (0.4) | 339 (0.4) | 13 (0.2) |
| First generation cephalosporin |  | 4,194 (4.5) | 3,637 (4.2) | 557 (8.7) |
| Second generation cephalosporin |  | 17,086 (18.3) | 16,379 (18.8) | 707 (11.1) |
| Third generation cephalosporin |  | 31,712 (33.9) | 30,253 (34.8) | 1,459 (22.9) |
| Third generation cephalosporin against psedomonas |  | 1,397 (1.5) | 1,321 (1.5) | 76 (1.2) |
| Fourth generation cephalosporin against psedomonas |  | 3,474 (3.7) | 3,267 (3.8) | 207 (3.2) |
| Carbapenem |  | 19,083 (20.4) | 16,607 (19.1) | 2,476 (38.9) |
| Aminoglycoside |  | 1,406 (1.5) | 1,219 (1.4) | 187 (2.9) |
| Quinolone |  | 2,461 (2.6) | 2,256 (2.6) | 205 (3.2) |
| Tetracycline |  | 616 (0.7) | 575 (0.7) | 41 (0.6) |
| Macrolide |  | 446 (0.5) | 378 (0.4) | 68 (1.1) |
| Metronidazole |  | 107 (0.1) | 86 (0.1) | 21 (0.3) |
| Clindamycin |  | 1,012 (1.1) | 904 (1.0) | 108 (1.7) |
| Vancomycin |  | 1,161 (1.2) | 907 (1.0) | 254 (4.0) |
| Other anti–MRSA drugs |  | 502 (0.5) | 383 (0.4) | 119 (1.9) |
| Others |  | 223 (0.2) | 202 (0.2) | 21 (0.3) |
| Antimicrobials combination | Mono therapy | 77,766 (83.2) | 73,142 (84.0) | 4,624 (72.6) |
|  | Combination therapy without drug-resistant pathogens | 3,228 (3.5) | 2,779 (3.2) | 449 (7.0) |
| Atypical pneumonia coverage | | 3,493 (3.7) | 3,184 (3.7) | 309 (4.8) |
| Initially MRSA coverage |  | 1,647 (1.8) | 1,280 (1.5) | 367 (5.8) |
| Initially pseudomonas coverage | Mono therapy | 41,005 (43.9) | 37,176 (42.7) | 3,829 (60.1) |
|  | Antipseudomonal combination | 2,651 (2.8) | 2,241 (2.6) | 410 (6.4) |
| Initially MRSA & pseudomonas coverage |  | 1,185 (1.3) | 863 (1.0) | 322 (5.1) |
| Length of antibiotic treatment, days, median [IQR] |  | 10 [7–15] | 10 [7–14] | 12 [8–18] |
| Length of hospital stay, days, median [IQR] |  | 17 [10–31] | 17 [10–30] | 24 [14–41] |
| Length of ICU stay, days, median [IQR] |  | 0 [0–0] | NA | 3 [2–7] |
| In-hospital mortality, n (%) |  | 10,088 (10.8) | 9,318 (10.7) | 770 (12.1) |
| Atypical pneumonia coverage included quinolone, tetracycline, and macrolide | | | | |
| ICU: intensive care unit, IQR: interquartile range, NA: not available, MRSA: methicillin-resistant *Staphylococcus aureus* | | | | |

Table S5-3. The initial empiric antimicrobials of choice and outcomes among septic patients with abdominal infection

|  |  | Overall | Ward | ICU |
| --- | --- | --- | --- | --- |
| Antibiotics, n (%) |  | 187,639 | 162,013 | 25,626 |
| Age, year, median [IQR] |  | 75 [63–84] | 76 [63–84] | 74 [64–82] |
| Male, n (%) |  | 105,368 (56.2) | 90,640 (55.9) | 14,728 (57.5) |
| Comorbidity | Malignant tumor, n (%) | 56,736 (30.2) | 49,255 (30.4) | 7,481 (29.2) |
|  | Hypertension, n (%) | 41,629 (22.2) | 36,177 (22.3) | 5,452 (21.3) |
|  | Diabetes mellitus, n (%) | 33,165 (17.7) | 28,482 (17.6) | 4,683 (18.3) |
|  | Heart failure, n (%) | 18,159 (9.7) | 15,159 (9.4) | 3,000 (11.7) |
|  | Cerebrovascular disease, n (%) | 17,431 (9.3) | 15,136 (9.3) | 2,295 (9.0) |
|  | Ischemic heart disease, n (%) | 13,859 (7.4) | 11,424 (7.1) | 2,435 (9.5) |
|  | Chronic respiratory disease, n (%) | 12,165 (6.5) | 10,957 (6.8) | 1,208 (4.7) |
|  | Chronic renal failure, n (%) | 4,861 (2.6) | 3,871 (2.4) | 990 (3.9) |
| Vasopressor use |  | 23,941 (12.8) | 18,983 (11.7) | 4,958 (19.3) |
| RRT use |  | 8,911 (4.7) | 4,146 (2.6) | 4,765 (18.6) |
| Ventilator use |  | 19,136 (10.2) | 8,399 (5.2) | 10,737 (41.9) |
| Penicillin derivative |  | 4,235 (2.3) | 3,981 (2.5) | 254 (1.0) |
| Ampicillin/sulbactam |  | 5,414 (2.9) | 4,841 (3.0) | 573 (2.2) |
| Tazobactam/piperacillin, piperacillin |  | 35,899 (19.1) | 30,900 (19.1) | 4,999 (19.5) |
| Sulbactam/cefoperazone |  | 8,616 (4.6) | 8,067 (5.0) | 549 (2.1) |
| First generation cephalosporin |  | 6,288 (3.4) | 4,980 (3.1) | 1,308 (5.1) |
| Second generation cephalosporin |  | 51,691 (27.5) | 44,764 (27.6) | 6,927 (27.0) |
| Third generation cephalosporin |  | 27,036 (14.4) | 25,211 (15.6) | 1,825 (7.1) |
| Third generation cephalosporin against psedomonas |  | 1,577 (0.8) | 1,456 (0.9) | 121 (0.5) |
| Fourth generation cephalosporin against psedomonas |  | 6,571 (3.5) | 5,994 (3.7) | 577 (2.3) |
| Carbapenem |  | 62,973 (33.6) | 49,206 (30.4) | 13,767 (53.7) |
| Aminoglycoside |  | 7,361 (3.9) | 6,640 (4.1) | 721 (2.8) |
| Quinolone |  | 3,716 (2.0) | 3,265 (2.0) | 451 (1.8) |
| Tetracycline |  | 1,120 (0.6) | 1,033 (0.6) | 87 (0.3) |
| Macrolide |  | 1,150 (0.6) | 942 (0.6) | 208 (0.8) |
| Metronidazole |  | 1,149 (0.6) | 884 (0.5) | 265 (1.0) |
| Clindamycin |  | 3,183 (1.7) | 2,586 (1.6) | 597 (2.3) |
| Vancomycin |  | 2,171 (1.2) | 1,495 (0.9) | 676 (2.6) |
| Other anti–MRSA drugs |  | 1,222 (0.7) | 820 (0.5) | 402 (1.6) |
| Others |  | 1,281 (0.7) | 1,135 (0.7) | 146 (0.6) |
| Antimicrobials combination | Mono therapy | 146,802 (78.2) | 128,775 (79.5) | 18,027 (70.3) |
|  | Combination therapy without drug-resistant pathogens | 12,245 (6.5) | 10,571 (6.5) | 1,674 (6.5) |
| Atypical pneumonia coverage | | 5,875 (3.1) | 5,151 (3.2) | 724 (2.8) |
| Initially MRSA coverage |  | 3,357 (1.8) | 2,295 (1.4) | 1,062 (4.1) |
| Initially pseudomonas coverage | Mono therapy | 101,252 (54.0) | 83,641 (51.6) | 17,611 (68.7) |
|  | Antipseudomonal combination | 8,892 (4.7) | 7,346 (4.5) | 1,546 (6.0) |
| Initially MRSA & pseudomonas coverage |  | 2,614 (1.4) | 1,682 (1.0) | 932 (3.6) |
| Length of antibiotic treatment, days, median [IQR] |  | 11 [7–17] | 10 [7–16] | 13 [8–20] |
| Length of hospital stay, days, median [IQR] |  | 19 [12–33] | 18 [11–32] | 26 [16–43] |
| Length of ICU stay, days, median [IQR] |  | 0 [0–0] | NA | 3 [1–6] |
| In-hospital mortality, n (%) |  | 19,436 (10.4) | 16,516 (10.2) | 2,920 (11.4) |
| Atypical pneumonia coverage included quinolone, tetracycline, and macrolide | | | | |
| ICU: intensive care unit, IQR: interquartile range, NA: not available, MRSA: methicillin-resistant *Staphylococcus aureus* | | | | |

Table S5-4. The initial empiric antimicrobials of choice and outcomes among septic patients with bone and soft tissue infection

|  |  | Overall | Ward | ICU |
| --- | --- | --- | --- | --- |
| Antibiotics, n (%) |  | 63,163 | 56,084 | 7,079 |
| Age, year, median [IQR] |  | 75 [62–84] | 76 [62–84] | 70 [58–80] |
| Male, n (%) |  | 33,291 (52.7) | 29,156 (52.0) | 4,135 (58.4) |
| Comorbidity | Malignant tumor, n (%) | 11,979 (19.0) | 10,942 (19.5) | 1,037 (14.6) |
|  | Hypertension, n (%) | 15,530 (24.6) | 13,977 (24.9) | 1,553 (21.9) |
|  | Diabetes mellitus, n (%) | 15,852 (25.1) | 13,719 (24.5) | 2,133 (30.1) |
|  | Heart failure, n (%) | 9,361 (14.8) | 8,118 (14.5) | 1,243 (17.6) |
|  | Cerebrovascular disease, n (%) | 7,453 (11.8) | 6,656 (11.9) | 797 (11.3) |
|  | Ischemic heart disease, n (%) | 4,561 (7.2) | 3,916 (7.0) | 645 (9.1) |
|  | Chronic respiratory disease, n (%) | 4,351 (6.9) | 4,001 (7.1) | 350 (4.9) |
|  | Chronic renal failure, n (%) | 2,213 (3.5) | 1,892 (3.4) | 321 (4.5) |
| Vasopressor use |  | 7,294 (11.5) | 5,610 (10.0) | 1,684 (23.8) |
| RRT use |  | 2,268 (3.6) | 1,319 (2.4) | 949 (13.4) |
| Ventilator use |  | 6,099 (9.7) | 3,191 (5.7) | 2,908 (41.1) |
| Penicillin derivative |  | 3,426 (5.4) | 2,859 (5.1) | 567 (8.0) |
| Ampicillin/sulbactam |  | 2,987 (4.7) | 2,630 (4.7) | 357 (5.0) |
| Tazobactam/piperacillin, piperacillin |  | 10,421 (16.5) | 9,164 (16.3) | 1,257 (17.8) |
| Sulbactam/cefoperazone |  | 286 (0.5) | 263 (0.5) | 23 (0.3) |
| First generation cephalosporin |  | 14009 (22.2) | 12719 (22.7) | 1290 (18.2) |
| Second generation cephalosporin |  | 6,569 (10.4) | 5,976 (10.7) | 593 (8.4) |
| Third generation cephalosporin |  | 12,138 (19.2) | 11,145 (19.9) | 993 (14.0) |
| Third generation cephalosporin against psedomonas |  | 633 (1.0) | 589 (1.1) | 44 (0.6) |
| Fourth generation cephalosporin against psedomonas |  | 3,221 (5.1) | 3,041 (5.4) | 180 (2.5) |
| Carbapenem |  | 16,311 (25.8) | 13,225 (23.6) | 3,086 (43.6) |
| Aminoglycoside |  | 1,213 (1.9) | 1,005 (1.8) | 208 (2.9) |
| Quinolone |  | 1,394 (2.2) | 1,201 (2.1) | 193 (2.7) |
| Tetracycline |  | 878 (1.4) | 798 (1.4) | 80 (1.1) |
| Macrolide |  | 525 (0.8) | 416 (0.7) | 109 (1.5) |
| Metronidazole |  | 165 (0.3) | 104 (0.2) | 61 (0.9) |
| Clindamycin |  | 4,989 (7.9) | 3,986 (7.1) | 1,003 (14.2) |
| Vancomycin |  | 2,612 (4.1) | 1,999 (3.6) | 613 (8.7) |
| Other anti–MRSA drugs |  | 1,865 (3.0) | 1,388 (2.5) | 477 (6.7) |
| Others |  | 253 (0.4) | 216 (0.4) | 37 (0.5) |
| Antimicrobials combination | Mono therapy | 45,580 (72.2) | 41,604 (74.2) | 3,976 (56.2) |
|  | Combination therapy without drug-resistant pathogens | 63,31 (10.0) | 5,018 (8.9) | 1,313 (18.5) |
| Atypical pneumonia coverage | | 2,739 (4.3) | 2,364 (4.2) | 375 (5.3) |
| Initially MRSA coverage |  | 4,398 (7.0) | 3,329 (5.9) | 1,069 (15.1) |
| Initially pseudomonas coverage | Mono therapy | 29,144 (46.1) | 24,963 (44.5) | 4,181 (59.1) |
|  | Antipseudomonal combination | 2,082 (3.3) | 1,686 (3.0) | 396 (5.6) |
| Initially MRSA & pseudomonas coverage |  | 2,717 (4.3) | 1,908 (3.4) | 809 (11.4) |
| Length of antibiotic treatment, days, median [IQR] |  | 13 [8–22] | 12 [8–21] | 17 [10–30] |
| Length of hospital stay, days, median [IQR] |  | 23 [13–43] | 22 [13–42] | 32 [18–57] |
| Length of ICU stay, days, median [IQR] |  | 0 [0–0] | NA | 4 [2–8] |
| In-hospital mortality, n (%) |  | 5,841 (9.2) | 5,075 (9.0) | 766 (10.8) |
| Atypical pneumonia coverage included quinolone, tetracycline, and macrolide | | | | |
| ICU: intensive care unit, IQR: interquartile range, NA: not available, MRSA: methicillin-resistant *Staphylococcus aureus* | | | | |
